# Spatial distribution of trace elements (As, Cd, Ni, Pb) from PM_10_ aerosols and human health impact assessment in an Eastern European country, Romania

**DOI:** 10.1007/s10661-021-08931-4

**Published:** 2021-03-10

**Authors:** Katalin Bodor, Zsolt Bodor, Róbert Szép

**Affiliations:** 1 Faculty of Natural Sciences, Doctoral School of Chemistry, University of Pécs, st. Ifjúság 6, 7624 Pécs, Hungary; 2 Faculty of Economics, Socio - Human Sciences and Engineering, Department of Bioengineering, Sapientia Hungarian University of Transylvania, Libertății Sq. 1530104, Miercurea Ciuc, Romania; 3 Institute for Research and Development for Hunting and Mountain Resources, st. Progresului 35B, 530240 Miercurea Ciuc, Romania

**Keywords:** PM_10_, Trace elements, Health impact assessment, Principal component analysis, Risk analysis

## Abstract

In the present study, the concentrations of trace elements in PM_10_ were determined and analyzed at 115 monitoring stations in Romania throughout the period 2009–2018. The spatiotemporal distribution of trace element concentrations of PM_10_, the source apportionment and health impact assessment, was carried out. The results showed a very high multi-annual mean concentration for PM_10_ and trace elements as well. The multiannual average concentration of PM_10_ was higher by 29.75% than the World Health Organization recommendation. All studied air pollutants showed a decreasing trend during the studied years, showing with 17.84%, 50.21%, 43.36%, 11.27%, and 72.09% lower values for PM_10_, As-, Cd-, Ni-, and Pb-, respectively, due to environmental regulations. To assess the human health impact, the hazard quotient (HQ) and cancer risk (CR) were calculated using the health risk model developed by the US Environmental Protection Agency (EPA). The Cd and Ni might present a non-carcinogenic risk to both adults and children; however, the hazard quotient values are higher than the safe limit, with 9.53 and 1.93, respectively. In addition, our study results revealed that the inhalation of As, Cd and the dermal absorption of all studied trace elements were considered as the most important risk factors for developing cancer, especially in case of adults.

## Introduction

Atmospheric pollution is a growing issue worldwide, adversely affecting both the human health and the ecosystem as well (Castillo et al., 2017; Keresztesi et al., 2018; Szép et al., 2018). Many epidemiological studies argue that particulate matter (PM) has become a decisive parameter of air pollution (Jena & Singh, 2017) and therefore can play an essential role in the chemical composition of precipitation (Keresztesi et al., 2020b, 2019). Particulate matter (PM_10_) with an aerodynamic diameter varying between 2.5 and 10 µm has a significant impact on human health (Xing et al., 2016) during long-term exposure if the concentration is higher for an extended period than the acceptable limit defined by WHO (20 µg m^−3^) (WHO, 2019). Furthermore, fine particles, known as PM_2.5_, are more harmful to human health; for example, they can get into bloodstream. Besides, particulate matter originated from traffic emission is often potentially enriched with toxic trace elements such as As, Cd, Cr, Cu, Zn, Pb, and Ni (Hao et al., 2018). When the trace element concentration exceeds the critical threshold, it may have a potentially toxic effect on human health and the ecosystem (Jena & Singh, 2017). According to many recent studies, the inhalable elements associated with the trace elements have an increasing effect on lung and cardiopulmonary morbidity and mortality (Dunea et al., 2016; Xia & Gao, 2010). Three possible routes of exposure to the toxic elements can be distinguished: ingestion, inhalation, and skin absorption; hence, trace elements have the potential to accumulate in biological systems, especially in fatty tissue (Du et al., 2013). In the air quality standard, the European Union has set an annual limit value for PM_10_ (20 µg m^−3^), Pb (0.5 µg m^−3^), As (6 ng m^−3^), Cd (5 ng m^−3^), and Ni (20 ng m^−3^), respectively (WHO, 2019). Furthermore, the limit value for the protection of human health imposed by Romanian Law 104/2011 is 40 μg m^−3^ (Law 104/2011, 2011). According to the International Agency for Research on Cancer (IARC), trace elements can cause a wide range of health issues since Cd is carcinogenic, and Pb is probably carcinogenic to humans. The high concentration or extreme exposure of Pb can cause severe neurological and hematological disorders on the exposed population, mainly children, while Cd disrupts the normal function of the kidneys.

On the other hand, short-term exposure to high Ni concentration via inhalation can cause kidney and lung disorders such as pulmonary fibrosis and renal edema. Exposure to high levels via ingestion can lead to neurological disorders and gastrointestinal discomforts, including diarrhea, nausea, and vomiting. Moreover, long-term exposure through dermal contact may result in dermatitis, while inhalation can cause many respiratory diseases such as nasal and lung cancer (Manalis et al., 2005). Finally, Ni and Cd can modify the structure of DNA and proteins by damaging nucleic acid synthesis (Sah et al., 2019).

Anthropogenic activities, such as the combustion of fossil fuels, and coal-burning are the primary As- and Ni-emitting sources (Keresztesi et al., 2020a). However, the metallurgical industry and road transport are also essential sources and are responsible for Ni pollution in PM_10_. Furthermore, it is well documented that the inhalation and ingestion of As can cause central nervous system disorders and gastrointestinal effects as well (Manalis et al., 2005). As is a highly toxic metal, causing a number of diseases: headaches, vomiting, abdominal pain, and even death when it occurs in high concentration (Roy & Saha, 2002). Exposure to lower concentrations can lead to cardiovascular diseases, while long-term effects of the inhaled inorganic As can result in dermatological diseases. It should also be mentioned the toxic effects of Pb can interact with proteins and regulate the skeleton Ca absorption as well; hence, the level of Pb in bones will increase while the Ca level will decrease (Lanphear et al., 1998). Moreover, the inactivation of the key proteins can lead to the formation of reactive oxygen species (ROS); thus, excess ROS will inhibit cellular processes at various levels (Papanikolaou et al., 2005).

In Romania, only a few studies have focused on airborne particulate matter and their trace element concentrations; the most recent studies were published by Dunea et al. (2016) and Proorocu et al. (2014). Further analyses are still required in terms of human health risk assessment of heavy metals determined from atmospheric particulate matter (PM_10_).

The main objective of this study is to analyze the heavy metal spatial and temporal variations (As, Cd, Ni, Pb) from PM_10_ in Romania and to evaluate the hazard quotient (HQ) and cancer risk (CR) in case of children and adults, using three different exposure pathways: inhalation, ingestion, and dermal absorption.

## Study area

### Sampling site

The studied area is situated in the crossroads of Central, Eastern, and Southeastern Europe, lying between 43°and 49° N latitudes and 20° and 30° E longitudes. Romania has borders with non-European Union members, including Republic of Moldova and Ukraine to east and northeast, respectively, where the EU Air Quality Environmental Protection Regulation has not yet been implemented. According to the National Institute of Statistics, the population of Romania in 2016 was 19,295,859 inhabitants in an area of 238,397 km^2^ (Ursu et al., 2018). The topography of Romania is almost evenly distributed, among mountains, hills plateaus, and plains.

The studied area’s climate is temperate continental in transition with an oceanic climate, influenced by Scandinavian-Baltic weather, Mediterranean climate, and the Black Sea weather. Romania has four distinct seasons, with average annual temperature of 11 °C in the south and 8 °C in the north. The average temperature in January and June is 1.1 °C and 20.6 °C, respectively.

## Materials and methods

In the present study, the temporal and spatial variations of PM_10_ and the concentrations of associated trace elements As, Cd, Ni, and Pb were examined. During the studied period (January 2009–December 2018), 722,925 daily data were collected and analyzed from 115 monitoring stations and seven different regions (C-Central, NE-North East, NW-North West, S-South, SE-South East, SW-South West, W-West), except the Bucharest region, where the trace element concentrations were determined from the PM_2.5_ fraction.

The raw data regarding daily pollutant concentrations was obtained from the National Air Quality Monitoring Network (www.calitateaer.ro), and the main purpose was to process and statistically analyze and interpret the retrieved data.

The reference method for the measurement of As, Cd, Ni, and Pb concentrations was in compliance with the SR EN 14902 standard “Ambient air quality from the fraction of PM_10_ particulate matter.” According to this method, the air sample was aspirated through a pre-weighed filter, and the analyzed trace elements (As, Cd, Ni, Pb) were passed from the filter into concentrate nitric acid and hydrogen peroxide (30%) solution, and microwave disintegration of samples was carried out in a closed vessel. The presence and concentration of trace elements were determined from the obtained solution using inductively coupled plasma mass spectrometry (ICP-MS) (SR EN 14902, 2006). For the spatial distribution, the county and the regional average concentrations were used.

### Statistical analysis

In order to decipher the temporal and spatial differences between regions and counties, descriptive statistics, monthly and annual trends, box plot analyses, and choropleth heat maps by data wrapper were used. Time series analysis was carried out only for the validated daily data. The average region concentration was calculated by using the monitoring station daily data according to Table 1, and the country average was obtained from the mean of regionals concentrations.

**Table 1:** Monitoring stations in Romania

	As	Cd	Ni	Pb	PM_10_
C	AB_1,3_ MS_1,2_ SB_3,4_	AB_1,3_ BV_1,3,4_ MS_1,2_ SB_1_,_3,4_	AB_1,3_ BV_1,3,4_ MS_1,2_ SB_3,4_	AB_1,3_ BV_1,3,4_ MS_1,2,3_ SB_1,3,4_	AB_1,3_ BV_1,2,3,4,5_ CV_1_ HR_1_ MS_1,2,3_ SB_1,3,4_
NE	BC_1,2,3,5_	BC_1,2_ IS_1,2,4,5,6_ SV_2,3_	BC_1,2_ IS_1,4_	BC_1,2_ IS_1,4,6_ SV_2,3_	BC_1,2_ BT_1_ IS_1,2,4,6_ NT_1,3_ SV_1,2,3_ VS_1,2_
NW	CJ_1,2,3,5_	BH_1,2_ CJ_1,2,3,5_ MM_1,2,3,4,5_	BH_1_ CJ_1,2,3,5_ SM_1,2_	BH_1_ CJ_1,2,3,5_ MM_1,2,3,4,5_ SM_1,2_	BH_1,2_ BN_1_ CJ_1,2,3,5_ MM_1,2,3,4,5_ SJ_1_ SM_1,2_
S	AG_1,2,3,4,6_ DB_1,2_ IL_1,2_ PH_1,2,3,5,6_	AG_1,2,3,4,6_ CL_1,2_ DB_1,2_ IL_1,2_ PH_1,2,3,5,6_	AG_1,2,3,4,6_ DB_1,2_ IL_1,2_ PH_1,2,3,5,6_	AG_1,2,3,4,6_ CL_1,2_ DB_1,2_ GR_1,2,3_ IL_1,2_ PH_1,2,3,5,6_ TR_1,2_	AG_1,2,3,4,6_ CL_1,2,3_ DB_1,2_ GR_1,2,3_ IL_1,2_ PH_1,2,3,5,6_ TR_1,2,3_
SE	CT_1,3,4,7_ GL_1,2,3,4_ TL_1,2,3_	CT_1,3,5,7_ GL_1,2,3,4_ TL_1,2,3_	CT_1,3,4,5,7_ GL_1,2,3,4_ TL_1,2,3_	BR_1,3,4_ CT_1,3,4,5,7_ GL_1,2,3,4_ TL_1,2,3_	BR_1,2,3,4_ BZ_1,2_ CT_1,3,4,5,7_ GL_1,2,3,4_ TL_1,2,3_ VN_1_
SW	GJ_1,2,3_ MH_1_ VL_1_	DJ_1,3_ GJ_2,3_ MH_1_ OT_1_ VL_1_	DJ_1,3_ GJ_1,2,3_ MH_1_ VL_1_	DJ_1,3_ GJ_1,2,3_ MH_1_ OT_1_ VL_1_	DJ_1,2,3,5,6_ GJ_1,2,3_ MH_1_ OT_1_ VL_1_
W	CS_1,2,3,4_ TM_1,2,3,5,6_	CS_1,2,3,4,5_ HD_1,2,3,4,5_ TM_1,2,3,5,6_	CS_1,2,3,4,5_ HD_1,2,3,4,5_ TM_1,2,3,5,6_	CS_1,2,3,4,5_ HD_1,2,3,4,5_ TM_1,2,3,5,6_	AR_1,2,3_ CS_1,2,3,4,5_ HD_1,2,3,4,5_ TM_1,2,3,5,6_

The monitoring stations are named according to the county abbreviation

The national average background level was calculated using the first quartile (25^th^ percentile). To determine the interrelationship between the investigated trace elements nonparametric Spearman correlation analysis was carried out (Ri386 3.5.3.).

Spearman’s correlation rank analysis was utilized to investigate the possible relationships between trace elements and PM_10_ for seven different regions (C, NE, NW, S, SE, SW, W). Using this approach, the strength of the relationship between two variables can be determined; hence, it can be deduced from the correlation coefficients if the elements have common sources; high correlation suggests common source (Javed et al., 2015). The monthly average concentration values were used during correlation analysis. The correlation coefficients between the two elements were considered significant at *P* < 0.1 and *r* ≥ + 0.165, and *r* ≤ −0.165.

### HCA and PCA

In environmental studies, principal component analysis (PCA) and hierarchical cluster analysis (HCA) are the most commonly used multivariate statistical methods (Bahloul, 2020; Chalvatzaki et al., 2019). Thus, trace elements (As, Cd, Ni, Pb) originated from PM_10_ were classified by using the Minitab17 statistical software, hierarchical cluster analysis method (Centroid Linkage, correlation coefficient distance), and the results were presented in a dendrogram. The PCA is the most frequently used technique of multivariate statistical analysis, and it minimizes the dimension of the variable in a dataset utilizing a reduced number of variables (Azid et al., 2015). For the factor analysis, the IBM SPSS Statistics 22 program principal component analysis was used, and the Kaiser-Meyer-Olkin sampling adequacy measurement was applied (KMO) during the PCA.

### Health risk assessment of heavy metals from PM_10_

Using the health risk model developed by EPA, the chemical daily intake (*CDI*),  exposure concentration (*EC*), and dermal absorption dose (*DAD*) were calculated via inhalation, ingestion, and dermal contact (Table 2):

**Table 2:** Parameters used in cancer non-carcinogen risk assessment (EPA, 2004)

Abbreviation	Description	Values	
		Children	Adults
*C*	Metal concentration in PM_10_ (μg m^−3^);		
*IngR*	Ingestion rate (mg day^−1^)	250	100
*ER*	Exposure frequency (days year^−1^)	250	
*ED*	Exposure duration (years)	6	24
*BW*	Average body weight (kg)	15	70
*AT*	Averaging time non-carcinogen (day)	2190	8760
*AT*	Averaging time carcinogen (day)	2190	25,550
*CF*	Conversion factor (kg mg^−1^)	10^−6^	
*SA*	The skin surface area that contacts with the PM (cm^2^)	2800	3300
*AF*	Skin adherence factor for the airborne particulates (mg cm^−2^)	0.2	
*ABS*	Dermal absorption factor As	0.03	
*ABS*	Dermal absorption factor Cd, other elements	0.01	
*ET*	Exposure time (h day^−1^)	24	
*ATn*	Average time for non-carcinogens (h)	52,560	210,240
*ATc*	Average time for carcinogens (h)		613,200

1 $${CDI}_{ing}=(C\times IngR\times EF\times ED\times CF)/(BW\times AT)$$

2 $${EC}_{inh}=(C\times ET\times EF\times ED)/AT$$

3 $${DAD}_{derm}=(C\times SA\times AF\times EV\times ABS\times EF\times ED\times CF)/(BW\times AT)$$

where *CDI*_*ingest*_— the chemical daily intake via ingestion

*EC*_*inh*_*— *exposure concentration via inhalation

*DAD*_*derm*_*— *dermal absorption dose

According to EPA (2004), the oral slope factor (*SFo*), oral reference dose (*RfDo*), gastrointestinal absorption factor (*GIABS*), inhalation unit risk (*IUR*), and inhalation reference concentrations (*RfCi*) were used to calculate the non-carcinogenic risks (Table 3.).

**Table 3:** Parameters used in cancer non-carcinogen risk assessment (EPA, 2004)

	*SFo*	*RfDo*	*GIABS*	*IUR*	*RfCi*
	(mg kg^−1^ day^−1^)^−1^	(mg kg^−1^ day^−1^)^−1^		(µg m^−3^)^−1^	(mg m^−3^)
As	1.50 × 10^+0^	3.00 x10^–4^	1	4.30 × 10^–3^	1.50 × 10^–5^
Cd		1.00 x 10^–3^	0.025	1.80 × 10^–3^	1.00 × 10^–5^
Ni		1.10 x 10^–2^	0.040	2.60 × 10^–4^	2.00 × 10^–5^
Pb	2.80 × 10^–1^	3.50 x 10^–3^	1	1.20 × 10^–5^	

Due to different behaviors of the respiratory systems and their characteristics, the quantitative assessment was carried out separately for adults and children. Furthermore, risk assessment was analyzed separately for non-carcinogenic and carcinogenic effects. The non-carcinogenic risk was assessed by the hazard quotient (HQ) and the carcinogen effect by the cancer risk (CR). Heavy metal elements are known to easily enter the human body through inhalation, ingestion, and dermal contact; hence, the HQ and CR risks for heavy metals in PM_10_ were calculated for ingestion, inhalation, and dermal contact, using the following equations (EPA, 2004):

4 $${HQ}_{ing}=CDI/RfDo$$

5 $${HQ}_{inh}=EC/(RfCi\times 1000 \mu g {mg}^{-1})$$

6 $${HQ}_{derm}=DAD/(RfDo\times GIABS)$$

7 $${CR}_{ing}=CDI\times SFo$$

8 $${CR}_{inh}=IUR\times EC$$

9 $${CR}_{derm}=DAD\times (SFo/GIABS)$$

CR represents the increased probability of tumor diseases incident above the general average due to the impact of the carcinogenic compound’s effects. During the evaluation of CR, chemicals were considered as having carcinogenic risk when the CR values varied from 10^−4^ to 10^−6^, representing that the cancer development during a human lifetime (70 years) is 1/10,000 or 1/1,000,000, respectively. Values lower than 10^−6^ for individual chemicals and pathways show no cancer risks. Generally speaking, a cumulative cancer risk higher than 10^−4^ is not accepted, and the maximum tolerable value is 10^−5^ (EPA, 2004).

## Results

### Statistical analysis of PM_10_ and trace element concentrations

During the studied period (2009–2018), the average concentration of PM_10_ in Romania was found to be 25.95 ± 2.8 µg m^−3^. The multiannual lowest average PM_10_ concentration was measured in the western region (23.25 µg m^−3^), and the highest in the southwestern region (31.03 µg m^−3^), where the PM_10_ concentration was 1.55 times higher than the WHO’s acceptable limit. At the same time, in the northeastern region, this limit value was exceeded by 1.38 times. The multiannual average concentrations within the studied area ranged between 0.67 ± 0.32 ng m^−3^ for As, 0.59 ± 0.21 ng m^−3^ for Cd, 2.25 ± 0.79 ng m^−3^ for Ni, and 0.030 ± 0.025 µg m^−3^ for Pb, respectively (Table 4.). According to these observations, in particulate matter, the highest concentration was recorded in the case of Pb, while the lowest concentration was found in the case of Cd.

**Table 4:** Descriptive statistics on the spatial distribution of pollutants

		C	NE	NW	S	SE	SW	W	RO	Stdev
PM_10_(µg m^−3^)	25P	18.31	23.27	17.23	21.99	21.34	20.25	18.20	20.08	
	Av	24.13	27.70	24.47	27.09	23.95	31.03	23.25	25.95	2.80
	CI95	22.64–25.62	26.63–28.78	22.84–26.10	26.10–28.08	23.32–24.58	30.49–31.56	22.00–24.49		
As (ng m^−3^)	25P	0.63	0.22	0.05	0.58	0.37	0.02	0.48	0.34	
	Av	1.04	0.30	0.18	0.82	0.63	0.82	0.90	0.67	0.32
	CI95	0.89–1.18	0.28–0.33	0.012–0.37	0.75–0.88	0.55–0.71	0.78–0.86	0.77–1.03		
Cd (ng m^−3^)	25P	0.50	0.29	0.18	0.35	0.24	0.25	0.39	0.31	
	Av	0.92	0.43	0.48	0.50	0.35	0.65	0.81	0.59	0.21
	CI95	0.18–1.02	0.39–0.46	0.41–0.55	0.46–0.54	0.32–0.39	0.62–0.67	0.65–0.98		
Ni (ng m^−3^)	25P	2.51	1.66	1.29	0.95	1.39	0.52	0.91	1.32	
	Av	3.90	2.08	2.11	1.39	2.13	1.77	2.38	2.25	0.79
	CI95	3.59–4.21	1.97–2.19	1.85–2.37	2.28–1.50	1.94–2.32	1.71–1.82	2.07–2.69		
Pb (µg m^−3^)	25P	0.02	0.01	0.01	0.01	0.01	0.00	0.01	0.010	
	Av	0.074	0.017	0.060	0.014	0.014	0.014	0.019	0.030	0.025
	CI95	0.063–0.085	0.015–0.018	0.044–0.075	0.010–0.017	0.013–0.015	0.011–0.016	0.017–0.022		

*25P* 25th percentile, *Av.* average, *CI95* 95% confidence interval, *RO* country average, *stdev* standard deviation

### Temporal distribution of pollutants

**Fig. 1:**
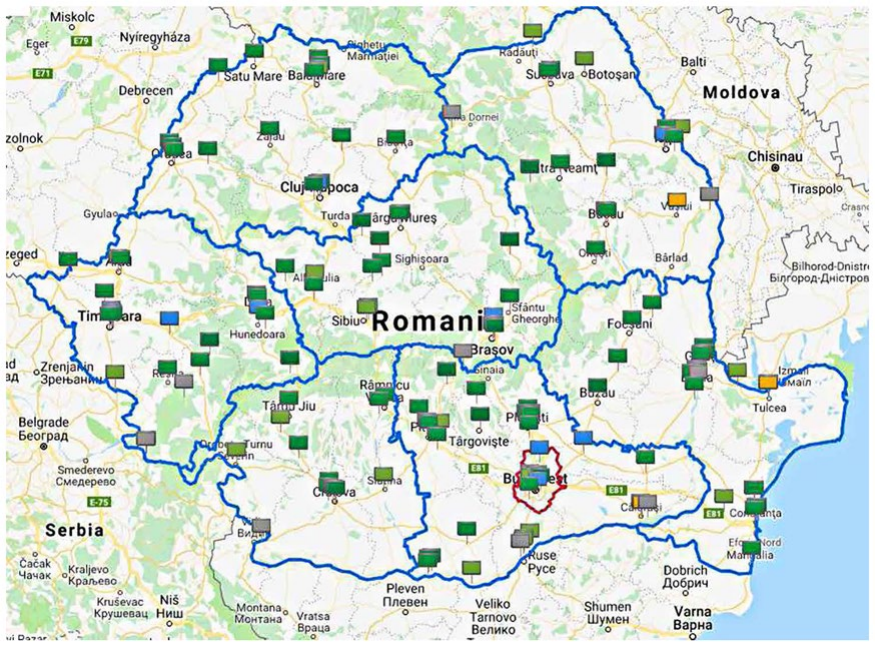
The geographic map of sampling sites. Stations are represented by small rectangles, and the different regions are separated by a blue line and Bucharest by a red line (www.calitateaer.ro)

**Fig. 2:**
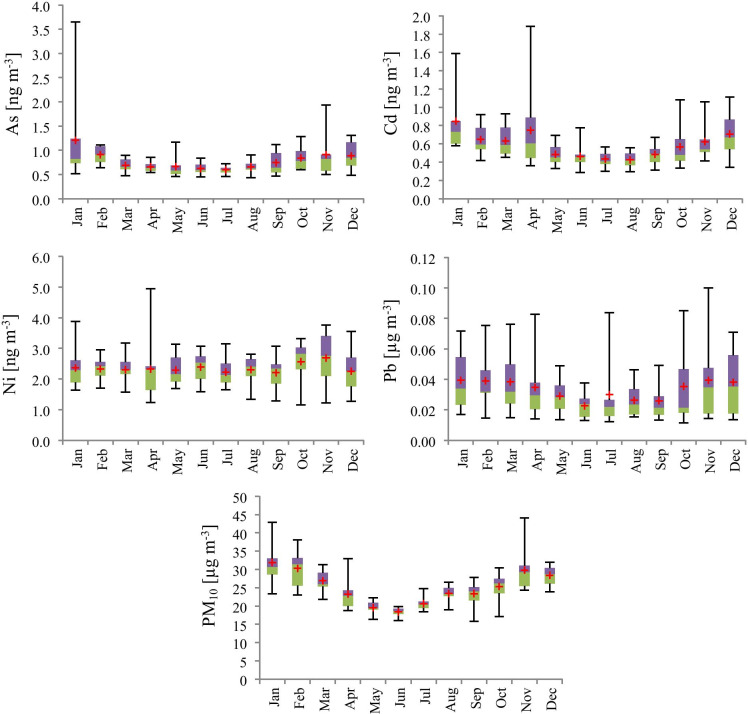
Box-plot analysis of multiannual monthly variations of pollutants The lower (green) and upper (purple) limits represent the first (25P) and third (75P) quartiles, means are represented by red crosses, and the ends of the whiskers represent the minimum and the maximum values

The multiannual monthly variation in PM_10_ and trace element concentrations across Romania are presented in Figs. 1 and 2. The seasonal pattern of PM_10_ and trace elements As, Cd, and Pb showed high concentrations in winter and low concentrations in summer, except for Ni, which shows an opposite trend.

**Fig. 3:**
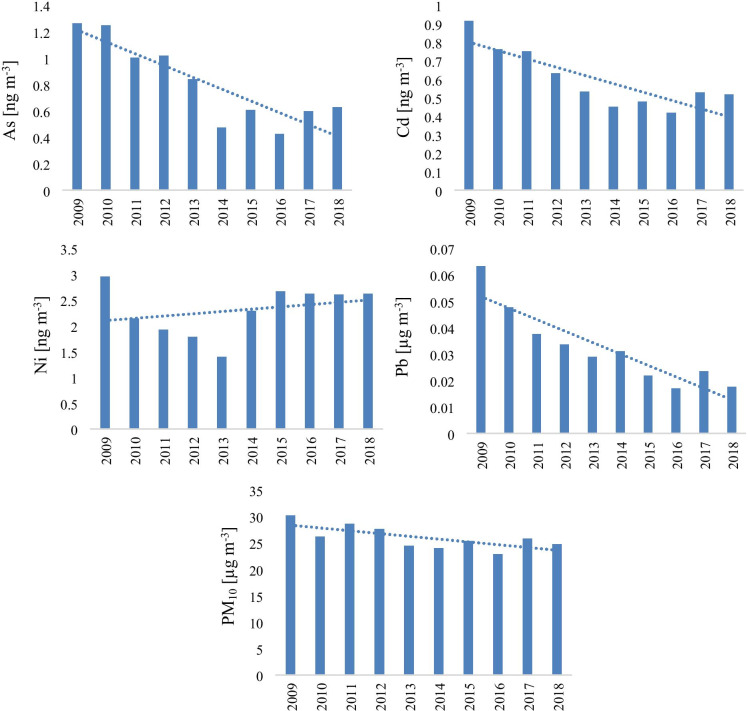
Annual trends of the concentrations of trace elements and PM_10_

Compared with the first reference year 2009, all the studied air pollutants showed a decreasing trend during the studied period. The decreasing percentage of the studied pollutants was 17.84% for PM_10_, 50.21% for As, 43.36% for Cd, 11.27% for Ni, and 72.09% for Pb (Fig. 3). Regarding the Ni variation, a decreasing trend was observed between 2009 and 2013; meanwhile, from 2014, a different (increasing) trend was detected.

### Spatial distribution of pollutants

**Fig. 4:**
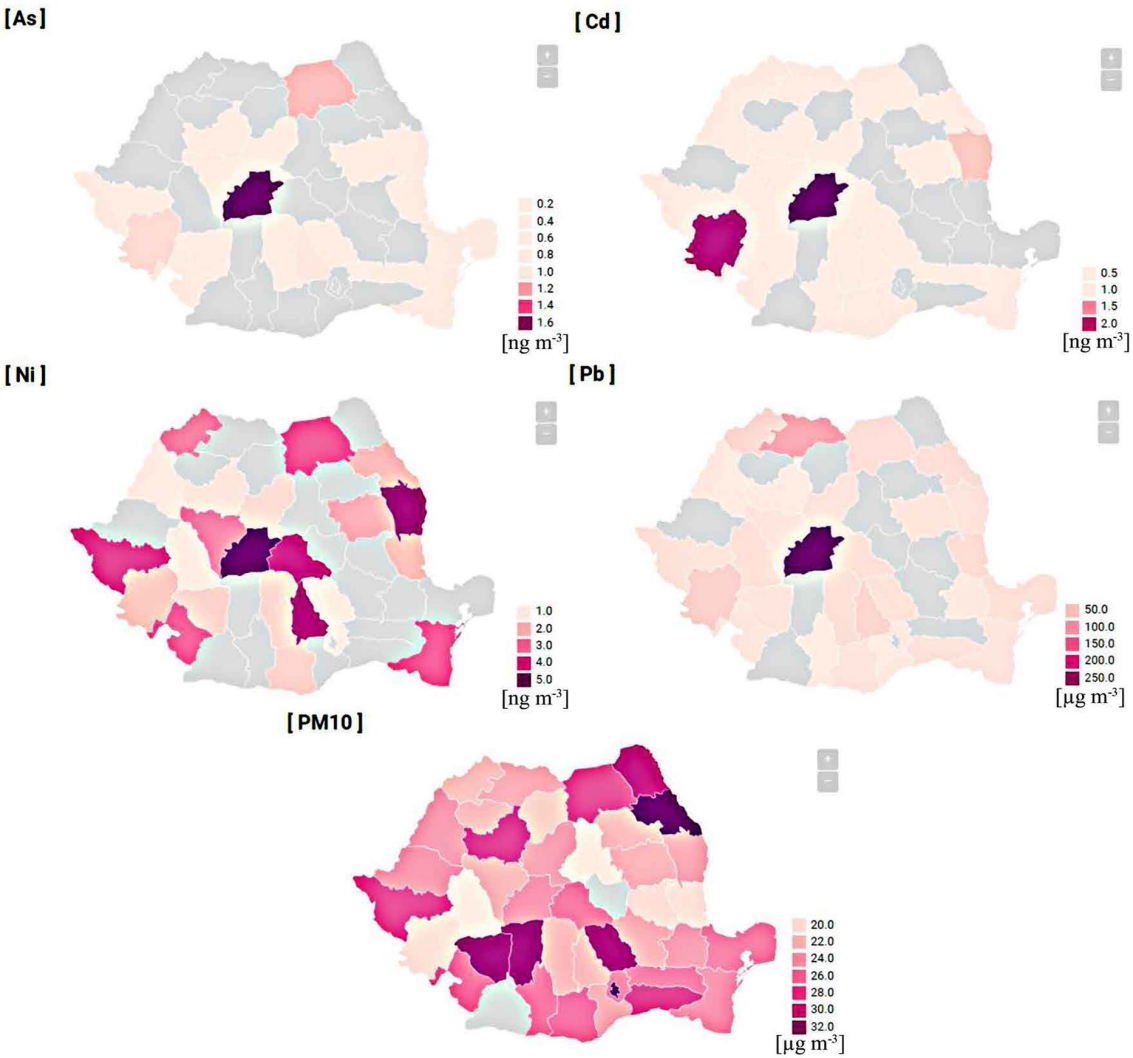
Spatial distribution of pollutants broken down by county

The total variation analysis between counties was assessed using the multiannual average concentration of each county, and the results are presented as heat maps by data wrapper (Fig. 4). The results revealed that the highest concentrations of trace elements were identified in Sibiu county, exhibiting 1.69 ng m^−3^, 2.32 ng m^−3^, 5.19 ng m^−3^, 0.292 ng m^−3^ for As, Cd, Ni, and Pb, respectively, which were mainly originated from one of the most polluted regions (Copșa Mică), while the highest particulate matter (PM_10_) concentration was in Iași county 33.61 µg m^−3^ (except Bucharest).

The percentage of exceedance days was calculated, when the measured values were higher than the WHO daily limit (50 µg m^−3^). Based on the Romanian average in the case of PM_10_, the daily exceedance was 5.3%, followed by Pb (1.05%), Cd (1.0%), As (0.56%), and Ni (0.42%), respectively. The highest exceedance rates were found in the central region Pb-4.53%, Cd-3.06%, As-1.77%, and Ni-1.48%, while the highest PM_10_ exceeding percentage (9.5%) was detected in the SW region (Table 5).

**Table 5:** Spatial distribution of exceedance percentages

	C	NE	NW	S	SE	SW	W	RO
PM_10_	5.74	5.57	4.76	4.78	2.56	9.50	4.15	5.30
As	1.77	0.00	0.07	0.39	0.32	0.64	0.70	0.56
Cd	3.06	0.00	0.59	0.45	0.18	0.86	1.88	1.00
Ni	1.48	0.01	0.03	0.28	0.38	0.00	0.79	0.42
Pb	4.53	0.02	2.39	0.08	0.01	0.29	0.06	1.05

The trace element concentrations measured in Romania were compared with those measured in different regions of the world; both the maximum value of the regions and the national average in Romania were included in the analysis as well (Table 6). It was found that the As concentration measured in Romania (1.1 ng m^−3^) was between the values of USA (Aneja et al., 2012) and Spain (Moreno et al., 2013). The Cd (0.97 ng m^−3^) concentration was much higher than in the USA, Spain, and Taiwan (Hsu et al., 2016), and the Ni concentration (3.94 ng m^−3^) was higher than what was reported in Spain. The Pb concentration in the central region (80 ng m^−3^) was close to the value of India (85.2 ng m^−3^).

**Table 6:** PM_10_ (µg m^−3^) and trace elements concentration (ng m^−3^) in different regions around the world

	Present study Central region	RO average	India	USA	Taiwan	Spain
As	1.10	0.67	8.9	0.839	3.39	1.56
Cd	0.97	0.59	6.6	0.1765	0.7	0.32
Ni	3.94	2.25	29.3	8.67	9.84	2.29
Pb	80	30	85.2	3.61	21.2	13.14
PM_10_	27.27	25.95	216	57.59	52.4	41.96
Source reference			Jena and Singh (2017)	Aneja et al. (2012)	Hsu et al. (2016)	Moreno et al. (2013)

### Correlation analysis of trace elements in different regions

**Fig. 5:**
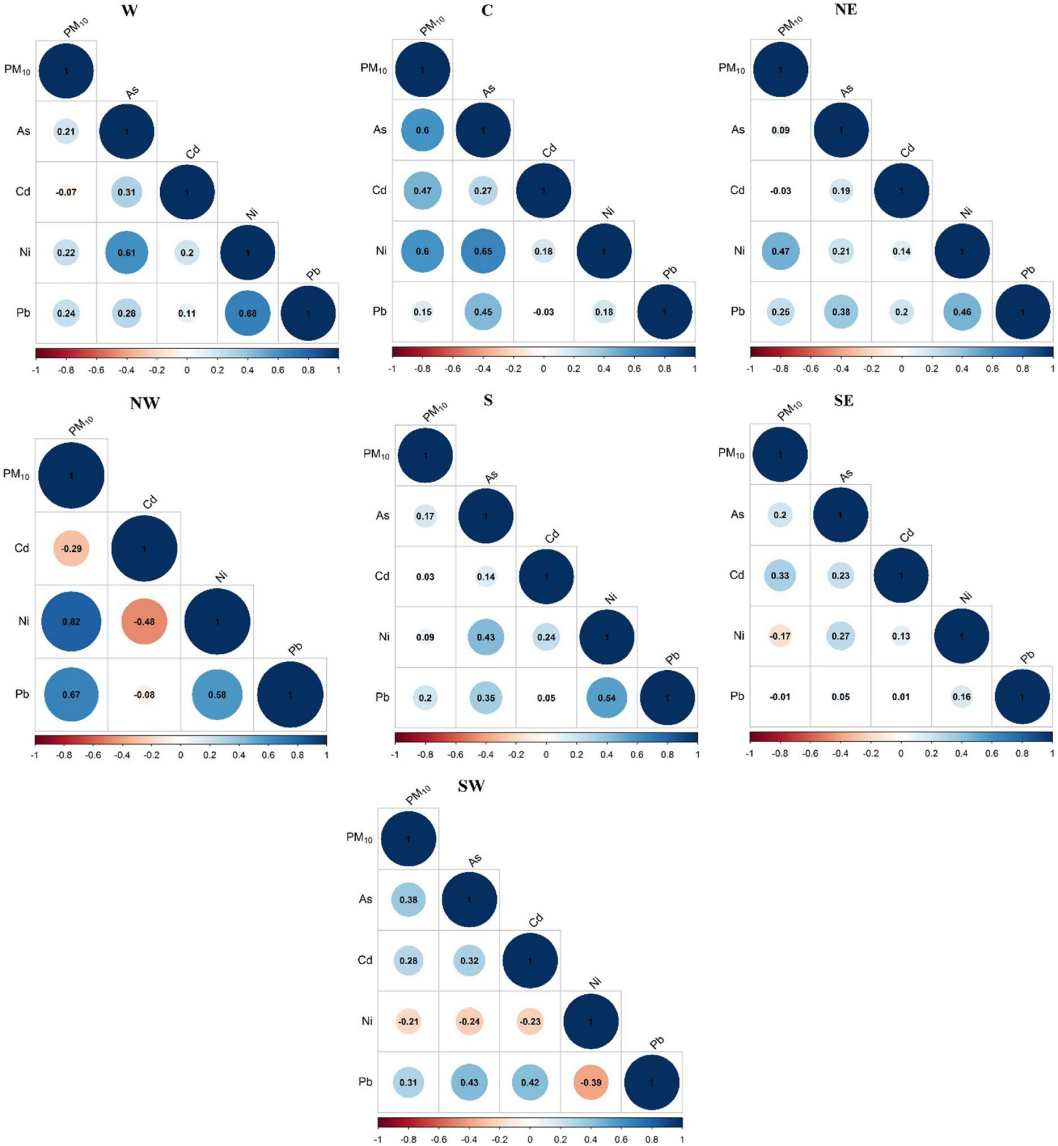
Spearman’s correlation coefficient matrices in different regions

 According to the results, a significant variation was observed in the correlation coefficients in different regions. The first three highest correlation levels were found between PM_10_ and Ni concentrations in the NW (0.82) > C (0.6) > NE (0.47) (Fig. 5.).

 Furthermore, a moderate correlation was identified between PM_10_ and Pb in the NW region (0.67). Regarding the PM_10_ and Cd interdependence, the concentrations showed a positive correlation in C (0.47) > SE (0.33) regions; however, negative correlation (−0.29) was detected in the NW region. A negative correlation may indicate foreign sources, and the regional distribution of pollutants is depending on the different climatological parameters as well. Analyzing the relationship between PM_10_ and As, the results revealed a significant positive correlation (0.6) in the central region. The relationship between the trace elements shows that the most significant correlation was obtained between Ni and Pb in the W (0.68) > NW (0.58) > S (0.54) > NE (0.46) > C (0.18) > SE (0.16) regions. A significant correlation was also observed between As and Pb in the central (0.45) > NE (0.38) > S (0.35) > W (0.28) regions, respectively. It should also be emphasized that there was a significant positive correlation between As and Ni in C (0.65) > W (0.61) > S (0.43) regions.

### Cluster and principal component analysis

**Fig. 6:**
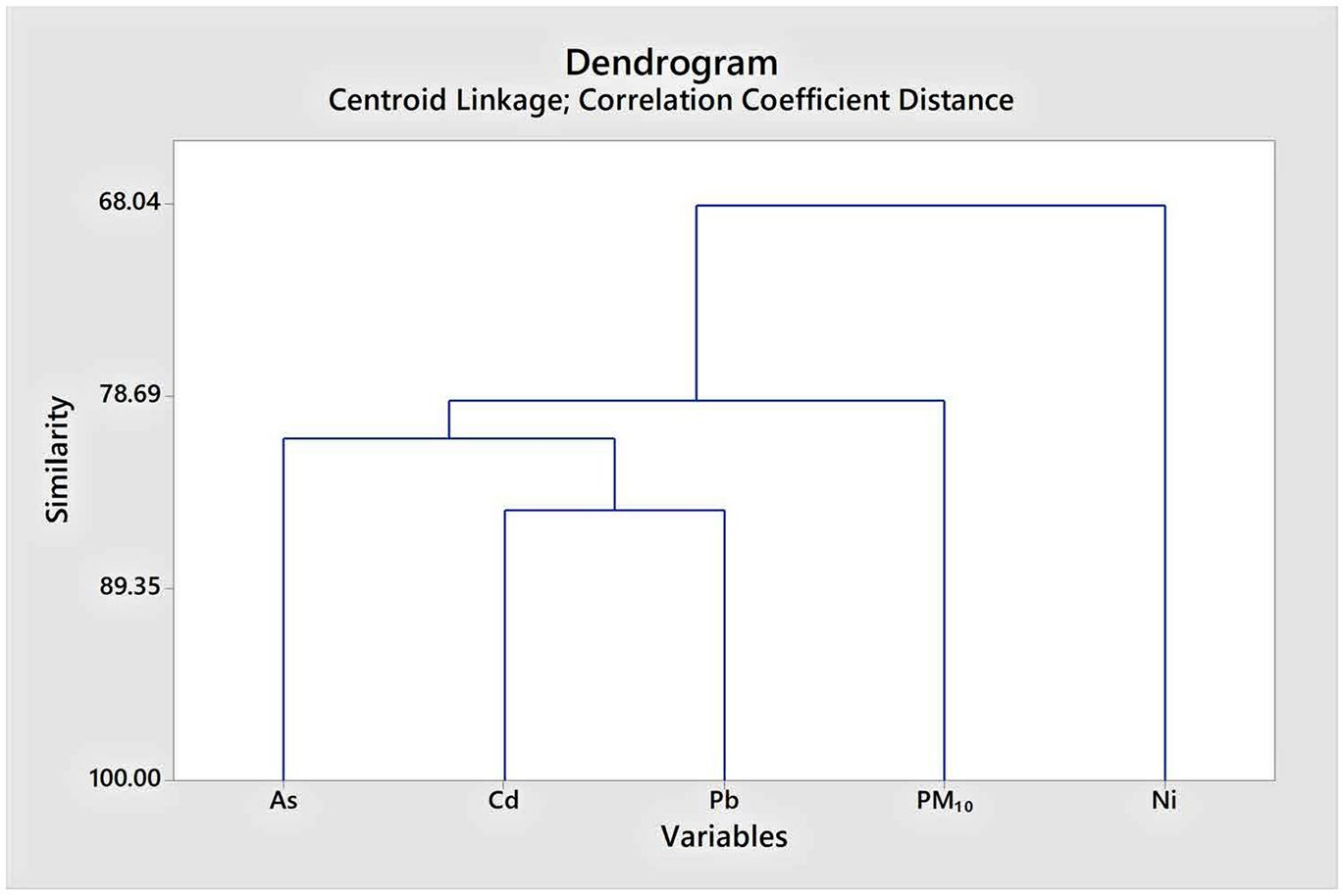
Cluster analysis of trace elements and PM_10_

Based on the average country concentrations, hierarchical cluster analysis was applied for As, Cd, Ni, and Pb to evaluate the potential contributing sources of heavy metals. According to the hierarchical cluster analyses, the variables (As, Cd, Ni, Pb, PM_10_) were classified in two different clusters. As, Cd, Pb, and PM_10_ are in the first cluster and Ni in a separate cluster (Fig. 6).

**Fig. 7:**
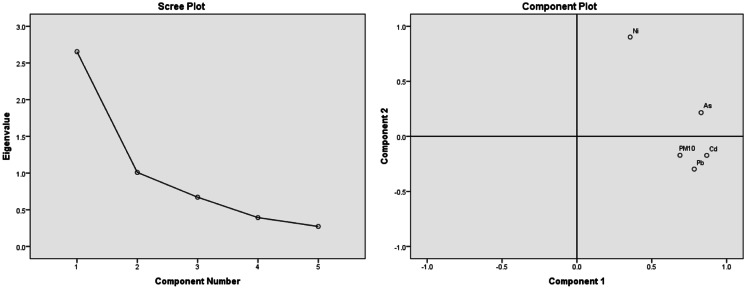
Principal component analysis scree plot (left) and component plot (right)

In order to decipher the origin as well as the possible common sources of heavy metals from the PM_10_ samples, principal component extraction was implemented. Two components were extracted from the component matrix, accounting for 73.27% of the total variance (Table 7; Fig. 7). Factor 1 contains As, Cd, Pb, and PM_10_ and represents 53.09% of the total variance.

**Table 7:** Component Score Coefficient matrix

Variable	Factor 1	Factor 2
As	0.312	0.0.214
Cd	0.327	−0.171
Ni	0.134	0.895
Pb	0.259	−0.295
PM_10_	0.259	−0.170
Eigen value	2.655	1.00
% variance	53.09	20.17
Cumulative % variance	53.09	73.27

The adequacy of the Kaiser-Meyer-Olkin (KMO) measure of sampling was 0.660, followed by the execution of the PCA, which means that the tested samples show medium adequacy.

### Health risk assessment

#### Non-cancer risk assessment

From the three studied exposure routes, a significant proportion of particulate trace elements pass into the human organs by inhalation. The calculated HQ values for the three studied exposure pathways were calculated and are presented in Table 8. We found that the HQ values were lower than the safe limit, via ingestion and dermal absorption for both studied groups (adults and children). Due to the fact that the different exposure routes were not available, the HQ values for Pb were not calculated.

**Table 8:** Hazard quotient (HQ) from trace elements in PM_10_ via ingestion, inhalation, and dermal contact for children and adults

	HQ_ing_		HQ_inh_		HQ_derm_	
	Children	Adults	Children	Adults	Children	Adults
As	1.95 × 10^–5^	2.09 × 10 ^−6^	7.01 × 10^–1^	7.01 × 10^–1^	1.93 × 10^–6^	3.51 × 10^–7^
Cd	5.30 × 10^–5^	5.68 × 10^–6^	9.53 × 10^+0^	9.53 × 10^+0^	6.99 × 10^–6^	1.27 × 10 ^−6^
Ni	1.93 × 10^–6^	2.07 × 10^–7^	1.92 × 10^+0^	1.92 × 10^+0^	1.60 × 10^–6^	2.90 × 10^–7^
Pb	8.61 × 10^–5^	9.23 × 10^–6^			2.84 × 10^–6^	5.17 × 10^–7^
HI	1.60 × 10^–4^	1.72 × 10^–5^	1.22 × 10^+1^	1.22 × 10^+1^	1.34 × 10^–5^	2.43 × 10^–6^

By inhalation, the HQ values were higher than the safe limit for both adults and children (= 1). The highest non-carcinogenic risk was detected for Cd (9.53). Ni also shows a non-carcinogenic risk with a value of 1.92. In contrast, the HQ exposure to As from the air particulate matter via inhalation shows a non-carcinogenic health risk because the calculated HQ value was lower than the safety limit (= 1). Taking into consideration, that the sum of the three trace elements (HI) reached the value of 1.22 × 10^1^ indicates non-carcinogen health risk for the mixture of trace elements via inhalation.

##### Cancer risk assessment

According to International Agency for Research on Cancer (IARC), the carcinogenic risk of the studied trace elements (As, Cd, Ni, Pb) in PM_10_ can be classified as carcinogens/probably, carcinogenic/possibly, and carcinogenic to human health. The carcinogenic risk via inhalation for adults in the case of As and Pb was higher than the permissible limit (1 × 10^–6^). The total cancer risk for adults and children was 4.01 × 10^–5^ and 1.00 × 10^–5^, respectively. The cancer risk of all PM_10_ bound trace elements via ingestion, inhalation, and dermal absorption for both adults and children is presented in Table 9. There are still notable differences between children and adults due to their different activities and behaviors (Khairy et al., 2011).

**Table 9:** Cancer risk (CR) from trace elements in PM_10_ via ingestion, inhalation, and dermal contact for children and adults

	CR_ing_		CR_inh_		CR_derm_	
	Children	Adults	Children	Adults	Children	Adults
As	7.51 × 10^–10^	3.22 × 10^–10^	3.88 × 10^–6^	1.55 × 10^–5^	1.73 × 10^–8^	9.26 × 10^–3^
Cd	2.77 × 10^–8^	1.19 × 10^–8^	1.47 × 10^–5^	5.88 × 10^–5^	2.13 × 10^–8^	1.14 × 10^–2^
Ni	3.10 × 10^–9^	1.33 × 10^–9^	8.54 × 10^–7^	3.41 × 10^–6^	2.38 × 10^–8^	1.27 × 10^–2^
Pb	7.23 × 10^–9^	3.10 × 10^–9^	5.58 × 10^–7^	2.23 × 10^–6^	5.55 × 10^–8^	2.97 × 10^–2^
SUM	3.88 × 10^–8^	1.66 × 10^–8^	2.00 × 10^–5^	8.00 × 10^–5^	1.18 × 10^–7^	6.31 × 10^–2^

Dermal absorption shows the highest cancer risk in the case of adults for all types of trace elements. Adults had a higher probability of experiencing carcinogenic risk compared with that of children due to the longer exposure time. However, there are more risk factors for cancer, which should be taken into consideration such as age, lifestyle, infections, contacts, etc. According to the calculations, in the case of As, the health risk for adults was greater via inhalation (1.55 × 10^–5^) than for children. The cancer risk for Cd was high for both adults and children, exhibiting 5.88×^−5^ and 1.47×^−5^, respectively. Via inhalation, the sum of all the elements under consideration increased to 2.00 × 10^–5^ for children and 8.00 × 10^–5^ for adults, indicating cumulative cancer risk due to inhalation exposure to the blend of elements. Finally, the cancer risk level of ingestion for all trace elements was within the acceptable range for both groups, children and adults, and the cumulative values were lower than the minimum acceptable level (1 × 10–^6^), implying negligible carcinogen risk.

## Discussions

Unfortunately, the air pollution in Romania is still an important issue to be addressed, and the implementation of the EU directives faces many challenges, and this is why the European Commission launched the first step in the infringement procedure against Romania for significant and persistent exceedances of limit values of fine particulate matter in the air (PM_10_, PM_2.5_), as well as for the inability to monitor air quality according to the EU laws (European Commision, 2019). In Romania, the main sources of air pollution come from the transport and energy sectors, especially from the use of fossil/solid fuels in households. Romania needs to make progress and significant steps in order to address this issue by restructuring the energy system and the household heating systems by favoring the integration of renewable energy sources and switching to natural gas or central heating. Furthermore, they need to invest more in infrastructure, traffic measures, and other traffic measures to control and prevent pollution resulted from the transport sector (European Commision, 2019).

The multiannual average concentration of PM_10_ in Romania was higher by 29.75% than the WHO’s acceptable limit (20 µg m^−3^) (WHO, 2019). The higher concentrations of PM_10_, As, Cd, and Pb in the cold period could be mainly attributed to domestic heating and other combustion sources, as well as to the stable meteorological conditions (low wind speed and static stability conditions) coupled to increased anthropogenic activities: biomass burning and home heating (Szép et al., 2019, 2017). The higher level of Ni concentration is attributable to increased industrial production and traffic flow during the summer (EMEP/EEA, 2019).

The annual decreasing trends could be explained in the light of the implementation of the European Union Environmental Protection regulation, which became applicable after joining the EU in 2007, modernization of the industrial sector, phasing out leaded petrol. On the other hand, the decreasing of Ni between 2009 and 2013 could be explained by the economic crisis; therefore, during that period, lower industry and transport activities were recorded, while the increasing trend (2014–2018) is in concordance with the economy recovery period (Santacatalina et al., 2012). Significant spatial differences were observed across the country, which depends strongly on the prevailing meteorological conditions and the proximity of emission sources (Elminir, 2005).

The high trace element concentrations measured in Caras Severin and Sibiu county are possible due to the waste resulted from the electrolytic refining of zinc, lead, and other heavy metals. Moreover, the highest infant mortality in Europe was reported in this region (Muntean et al., 2010). Many of the tailings dumps and tailings ponds are still un-rehabilitated, causing a significant impact on the environment. High concentrations of Cd were also detected in Caras Severin county, where the ferroalloys and cast iron manufacturers as well as the presence of large thermal power plants in the area are responsible for the elevated Cd pollution (2.35 ng m^−3^) (Caraş-Severin County Council, 2020). The possible reason why the highest PM_10_ concentration was measured in Iasi county is due to the fact that it is bordered by non-EU member countries, where the Air Quality Environmental Protection Regulation has not yet been implemented.

On a pollution scale, Romania is in the middle, after India and Taiwan, and before the USA and Spain. The intensity of human (anthropogenic) activities is responsible for the variation of heavy metal concentration from PM_10_ around the world, including traffic and industrial emissions, land use patterns, and local meteorological conditions as well (Al-Khashman, 2007; Doabi et al., 2017).

The positive Spearman correlation found between the studied pollutant can indicate that the trace elements are mostly derived from the same sources, generally originating from anthropogenic activities, vehicle emissions, metal corrosion (Cd) (Alam et al., 2014), and coal burning (As, Cr, Pb) as well (Tian et al., 2010; Zhang et al., 2009). According to PCA results, As, Cd, and Pb are coming from different sources (mixing sources), such as vehicle exhaust, coal burning, diesel fuel, lubrication oil, tire and brake wearing, mining, and industrial emissions (Foti et al., 2017; Mansha et al., 2012), while the Ni could be derived from vehicle exhaust through oil burning (Fang et al., 2017; Wang et al., 2013). On the other hand, the Pb sources could be originated from the soil dust (Zhang et al., 2016). In Romania, the main sector responsible for the Ni emission is the energy and fuel production and processing, while for Pb, the heavy industry (“https://www.eea.europa.eu/data-and-maps/dashboards/air-pollutant-emissions-data-viewer-3,” n.d.).

Based on the calculated human health effect in case of inhalation, the hazard quotient values were higher than the safe limit for Cd and Ni. Furthermore, the results revealed that the inhalation and dermal absorption of trace elements represent a serious risk of cancer.

## Limitations

In this study, trace elements As, Cd, Ni, and Pb were analyzed from PM_10_ fractions, and the resulted specific data was made publicly available; hence, all data were obtained from the National Air Monitoring System. Permanent air monitoring stations are implemented all over the country to monitor air quality, since all countries from the EU, including Romania, have an EU obligation to report regularly the air pollution levels. The current approach has only informative and indicative character. For example, the public health consequences of air pollution usually are analyzed by using only a single-pollutant approach; however, air masses always contain many pollutants in different amounts; therefore, in order to decipher the health risk of a complex mixture of air pollutants, the multi-pollutant approaches are desirable. Epidemiological studies are key instruments in establishing the impact of air pollution on human health in the combination with other confounding factors. Since, air pollutants do have adverse health effects at certain levels, further epidemiologic studies are necessary to analyze and quantify the consequences of long-term exposure especially in the case of air pollutants at low concentrations and dose/response effects as well.

## Conclusions

The present study has attempted to analyze the concentration and spatial distribution patterns of trace elements in PM_10_, and the human health risks were calculated for each trace element.

During the studied period, significant decreasing trends were detected in the concentrations of PM_10_, As, Cd, Ni, and Pb. Since 2007, Romania has been an EU member, and several environmental protection regulations were implemented through the years; however, many environmental pollution issues have yet to be solved. Despite the reduction trend, the multiannual mean concentration is still higher than the required values. The results showed significant differences in the PM_10_ and trace element concentrations and revealed a clear seasonal variation in PM_10_ concentrations, with the highest concentrations during the winter season and the lowest in summer. The multiannual minimum and maximum PM_10_ concentration was measured in the W (23.25 µg m^−3^) and SW region (31.03 µg m^−3^). The highest trace element concentration determined from the PM10 was recorded in the case of Pb, while the lowest concentration was found in the case of Cd. From the health risk calculations, inhalation was the most dominant exposure route for humans to intake the particle-bounded trace elements. According to the health risk analysis results, during exposure to the mixture of trace elements, the sum of Hazard Index (non-cancer risk) showed an elevated level. Via inhalation and dermal absorption, the potential carcinogenic risk was detected exceeding the carcinogen-acceptable level (1 × 10^–6^), indicating an increased risk of cancer in adults in the studied area. Due to the longer exposure, adults had a higher probability of experiencing carcinogenic risk than children. Considering the missing data issue, the situation could be much more serious in reality than the reported health risk assessment by toxic metals in PM_10_. In this context, Romania needs to improve its environmental protection measures and procedures, to reduce air pollution, mainly in the crowded cities and in industrialized regions; thus, further research is required to evaluate the human health effect in the heavily industrialized region in Romania.
